# Case Report: *GNAQ*- and *SF3B1* Mutations in an Aggressive Case of Relapsing Uveal Ring Melanoma

**DOI:** 10.3389/fonc.2022.873252

**Published:** 2022-05-25

**Authors:** Michelle Prasuhn, Josephine Christin Freitag, Sabine Lüken, Vinodh Kakkassery, Hartmut Merz, Almuth Caliebe, Malte Spielmann, Mahdy Ranjbar, Felix Rommel

**Affiliations:** ^1^ Department of Ophthalmology, University Hospital Schleswig-Holstein, University of Lübeck, Lübeck, Germany; ^2^ Hämatopathologie Lübeck, Reference Centre for Lymph Node Pathology andHematopathology, Lübeck, Germany; ^3^ Institute of Human Genetics, University Hospital of Schleswig-Holstein, University of Lübeck, Lübeck, Germany

**Keywords:** uveal melanoma, ring melanoma, GNAQ, SF3B1, brachytherapy, case report

## Abstract

The molecular mechanisms for uveal ring melanoma are still unclear until today. In this case report, we describe a patient with a malignant uveal melanoma with exudative retinal detachment that had been treated with plaque brachytherapy, resulting in successful tumor regression. After 1 year, a ring-shaped recurrence with extraocular extension appeared, and the eye required enucleation. Histological and molecular genetic analyses revealed an epithelioid-cell-type melanoma with complete circumferential involvement of the ciliary body and, so far, unreported GNAQ and SF3B1 mutations in ring melanoma. Therefore, this report gives new genetic background information on this ocular tumor usually leading to enucleation.

## Introduction

Uveal melanomas represent the most frequently diagnosed primary intraocular malignant tumor in adults and are potentially life-threatening (1). Among uveal melanomas, the variant ring melanoma is a very rare entity and comprises around 0.3% of all uveal melanomas (2). It was first introduced as “ring sarcoma” by Ewetzky *et al.* to describe an unusual circumferential growth pattern of uveal melanoma involving the ciliary body and the iris (3). Ring melanomas are summarized as uveal melanomas manifesting as a circumferential growth pattern around the eye that can present in the choroid, ciliary body, anterior chamber angle, and iris with different features (2).

Here we describe a rare case of ring melanoma that manifested as a relapse of a uveal melanoma after plaque brachytherapy. The molecular genetic assessment revealed a unique and, so far, unreported mutation status.

## Background

### Patient Information

A 72-year-old female patient was referred to our clinic with a suspicious, prominent choroidal lesion on her left eye that was noticed by her ophthalmologist in a routine checkup. The patient did not report any symptoms, and further ophthalmological medical history was unremarkable. General medical history revealed type 2 diabetes and hypertension treated with metformin, hydrochlorothiazide, nitrendipine, and candesartan. The patient did not report any prior malignancies, and the family health history concerning cancer or ophthalmological conditions was unremarkable as well.

### Case Presentation

**Figure 1:**
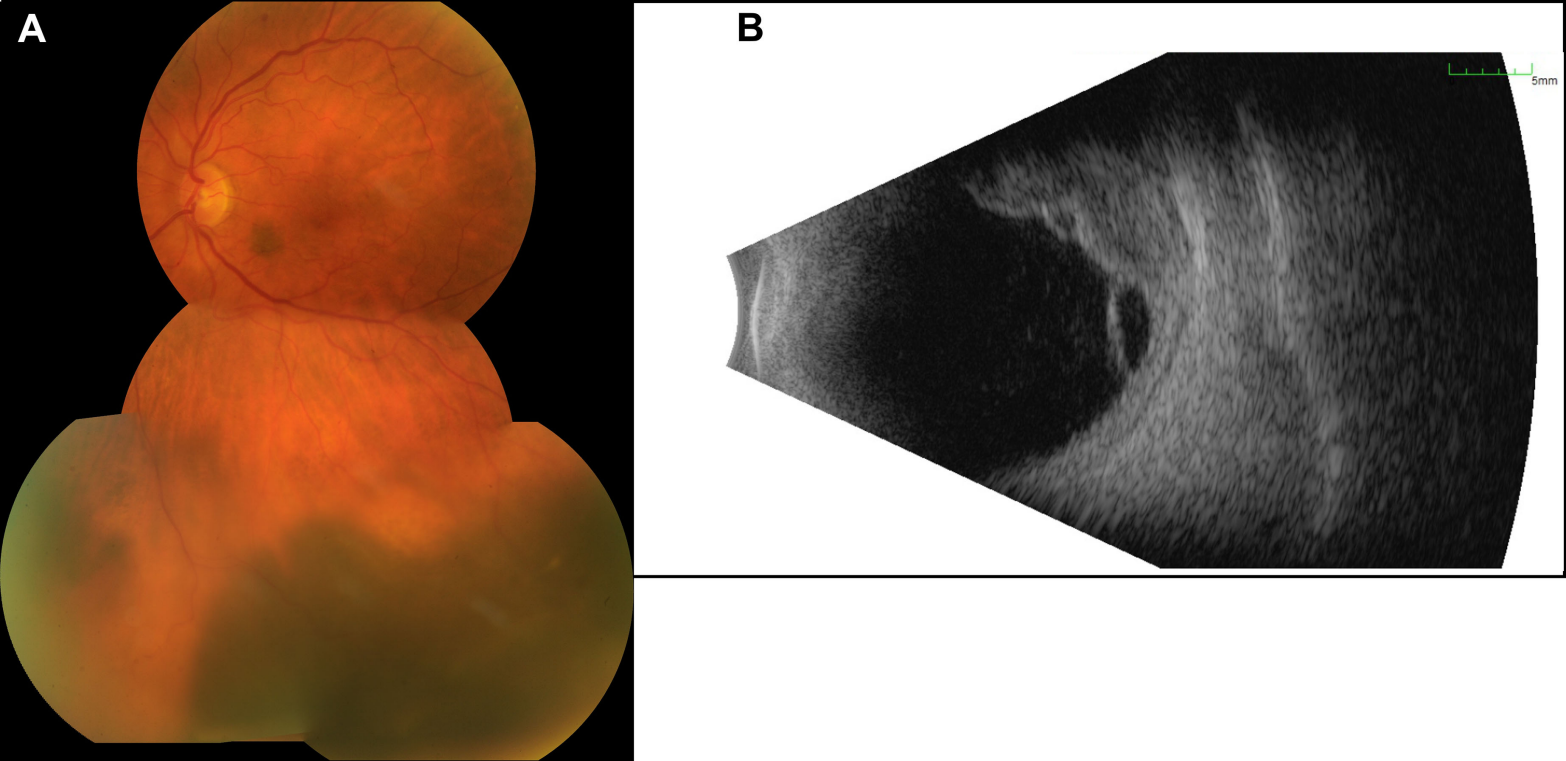
Presentation of the initial tumor. **(A)** The image of the posterior pole illustrates a melanotic tumor with subretinal fluid. **(B)** The B-scan ultrasound reveals a multilobulated tumor with a size of 13.6 × 10.8 mm and a thickness of 6.0 mm and with surrounding subretinal fluid.

The best-corrected visual acuity (BCVA) on both eyes was 20/30, and the intraocular pressure was normal (15/18 mmHg). Upon slit-lamp examination, we found a moderate cataract on both eyes and an otherwise normal anterior segment, including any alterations of the iris. Fundoscopy of the left eye revealed a prominent melanocytic proliferation in the inferior hemisphere with surrounding subretinal fluid extending towards the inferior arcade (**Figure 1**). Another small, flat melanocytic lesion was visible next to the optic disk. Using ultrasound B-scans, we recognized a medium reflective tumor with a base of 13.6 × 10.8 mm and a thickness of 6.0 mm, with surrounding tumor-related retinal detachment (**Figure 1**). We carried out an ultrasound biomicroscopy (UBM), which showed a little swelling of the inferior ciliary body (max. 2.5 mm) but no infiltration of the surrounding structures. Upon these findings, the clinically suspected diagnosis was a uveal melanoma. Staging analyses including magnetic resonance imaging of the head, computed tomography (chest and abdomen), and ultrasound of the abdomen (dermatological and gynecological assessment) revealed no metastases or other suspicious lesions. The blood tests were unremarkable, especially for liver enzymes and S100. We discussed with the patient the option of taking a biopsy before therapy initiation, but since the tumor showed clinically distinct features for uveal melanoma, we decided on immediate therapeutic intervention. Therefore, we performed brachytherapy of the tumor with ruthenium-106 only shortly after the first visit. Intraoperatively, the diaphanoscopy showed that the tumor expanded even further anteriorly and along the ciliary body than we preoperatively suspected in UBM. Therefore, we first applied the ruthenium-106 plaque above the main part of the tumor (tumor apex dose 150 Gy, scleral dose 1,004 Gy) and relocated the plaque “edge to edge” after 7 days and above the minor part for another 6 days (tumor apex dose, 125 Gy; scleral dose, 850). During plaque removal, we additionally applied triamcinolone intravitreally to reduce the subretinal fluid surrounding the tumor as previously described by Parrazzoni *et al.* (4).

**Figure 2:**
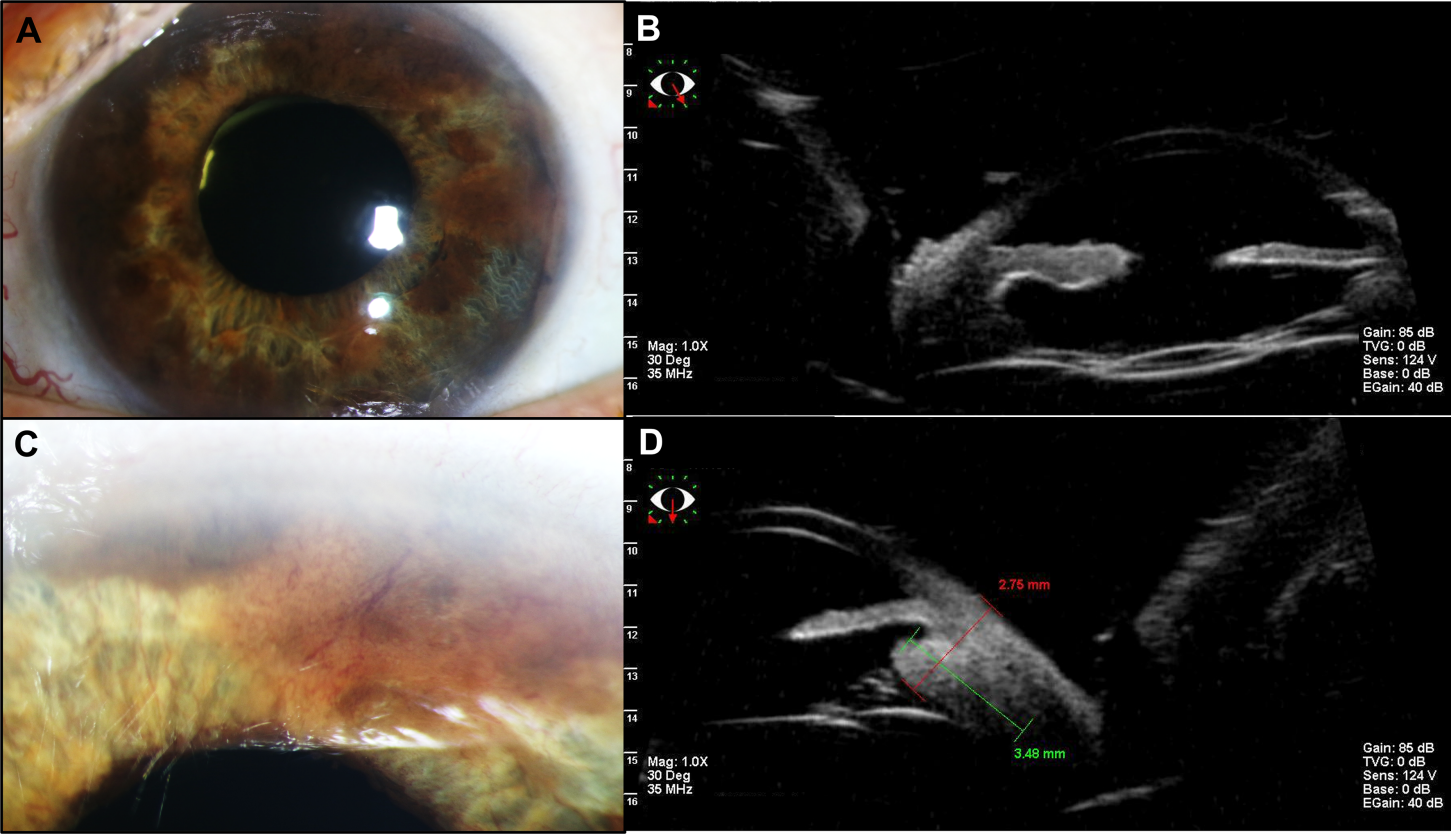
Presentation of the recurrence. **(A)** The slit-lamp examination of the anterior segment shows an episcleral sentinel vessel and vascularized, melanotic lesions infiltrating the iris circumferentially. **(B)** Close-up image focusing on the iridial lesions with vascularization. **(C)** The ultrasound biomicroscopy (UBM) of the temporal–inferior iridociliary junction illustrates the thickening of the iris. **(D)** The UBM of the inferior–anterior segment reveals ciliary body infiltration, measuring 2.75 × 3.84 mm at this site. With UBM, iridial and ciliary body thickening was unveiled circumferentially.

**Figure 3:**
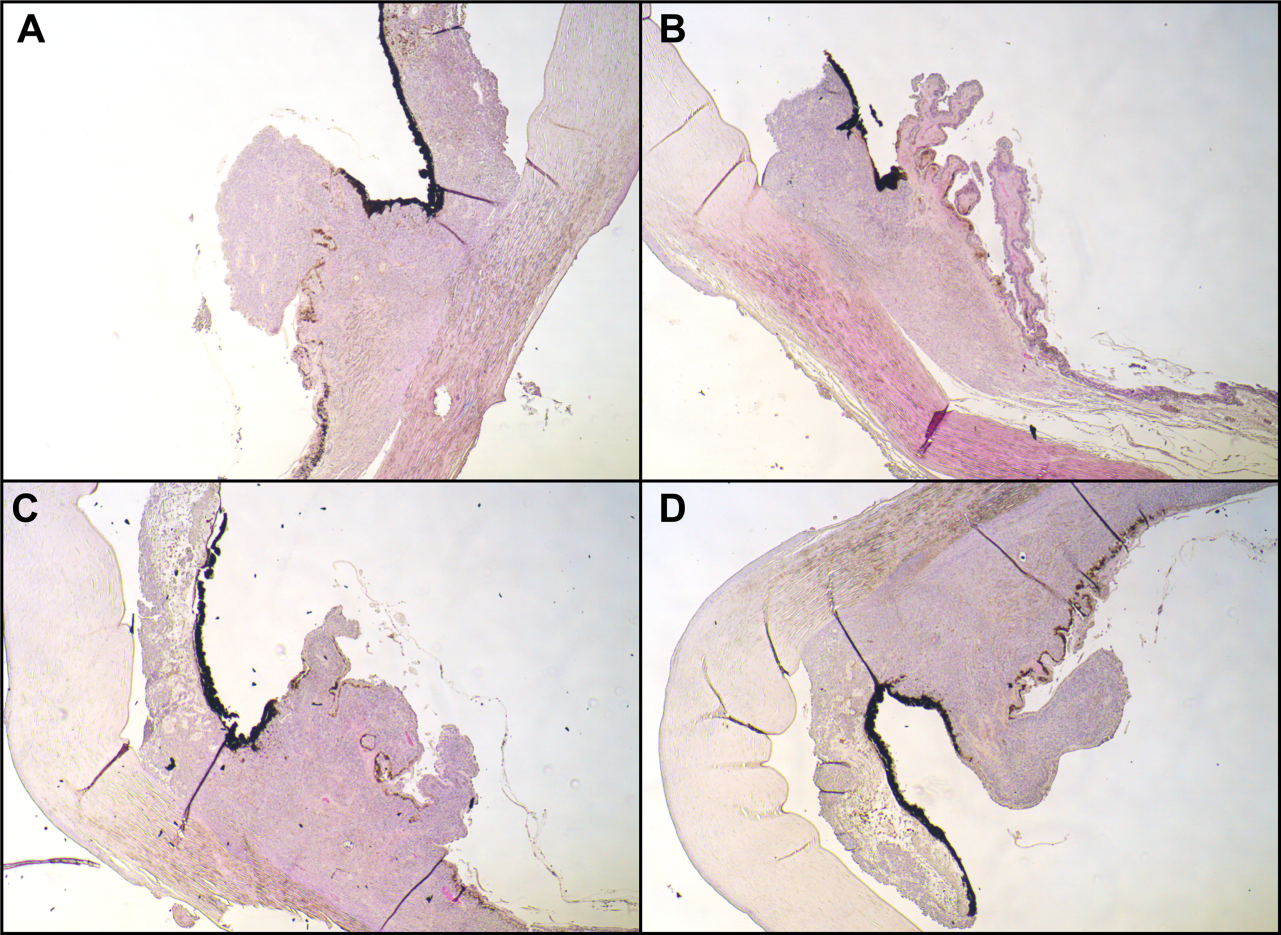
Histopathological examination. Hematoxylin and eosin stain of the iridociliary junction, magnification: ×10. **(A)** Superior, **(B)** nasal parts of the iris were missing due to paraffin embedding; **(C)** inferior; **(D)** temporal. All images expose infiltration of an epithelioid-cell-type tumor infiltrating the ciliary body.

In the subsequent visits over 6 months, we noted a continuous decrease in tumor size until there was no uveal thickening detectable on ultrasound and no more subretinal fluid. At 7 months after the therapy with ruthenium-106, we confirmed a good local tumor control. However, the patient developed a radiation retinopathy with macular edema and deterioration of BCVA to 20/80, so we decided to inject triamcinolone intravitreally. When we saw the patient in our clinic 4 weeks after the injection, she had developed a posterior pole cataract which decreased the vision to hand motion and did not enable us to perform a fundus examination anymore. Therefore, we soon carried out cataract surgery without complications, which improved the patient’s vision. Only a few weeks afterward, we noticed a suspicious melanotic lesion of the iris with vascularization that was not present before. The UBM revealed thickening of the ciliary body, measuring 1.5 × 1.5mm. Furthermore, we saw a still active radiation retinopathy, and a new exudative retinal detachment has formed. Suspecting a toxic tumor syndrome, we decided to perform a vitrectomy with endoresection of the original melanoma, reattachment of the retina with silicone oil tamponade, and biopsy of the new iridial lesion. The latter unveiled histopathological findings that are typical of uveal melanoma with an epithelioid cell type (increased expression of HMB45 and MelanA and high proliferative activity in Ki67 staining). With the biopsy of the iris and the resected melanoma, we carried out additional molecular genetic analyses. Fluorescence *in situ* hybridization (FISH) of the iris biopsy disclosed monosomy 3, heterozygous deletion in 1p36, and excessive copies of *MYC* in 8q24. In next-generation sequencing [the targeted gene panel including *GNA22*: codon p.Arg625 and p.Gln209 (NM_002067); *GNAQ*: codon p.Arg183 and p.Gln209 (NM_002072); *SF3B1*: codon p.Arg625 (NM_012433) and MiSeq sequencing by synthesis (Illumina; coverage 250, average reads per exon >1,000), both probes revealed c.626A>T in *GNAQ* (encoding G Protein Subunit Alpha Q), leading to p.Gln209Leu, and c.1874G>A in *SF3B1* (encoding splicing factor 3B subunit 1), leading to p.Arg625His. Analyses concerning *GNA11* (encoding G Protein Subunit Alpha 11) showed a wild-type status. Soon after vitrectomy and endoresection, we noticed that the iridial lesions had increased in number and size, along with severe iridial vascularization and a prominent episcleral (sentinel) vessel (**Figure 2**). In UBM, we saw a circumferential infiltration of the iris and ciliary body, leading to the diagnosis of a ring melanoma (**Figure 2**). Considering the aggressiveness of the tumor progression to a ring melanoma and taking into account the toxic tumor syndrome with vision loss to hand motion, we had to decide on the enucleation of the left eye in agreement with the patient. The enucleation took place without complications, and the histopathological analyses of the enucleated eye confirmed the annular growth of the tumor around the iris (**Figure 3**).

**Figure 4:**
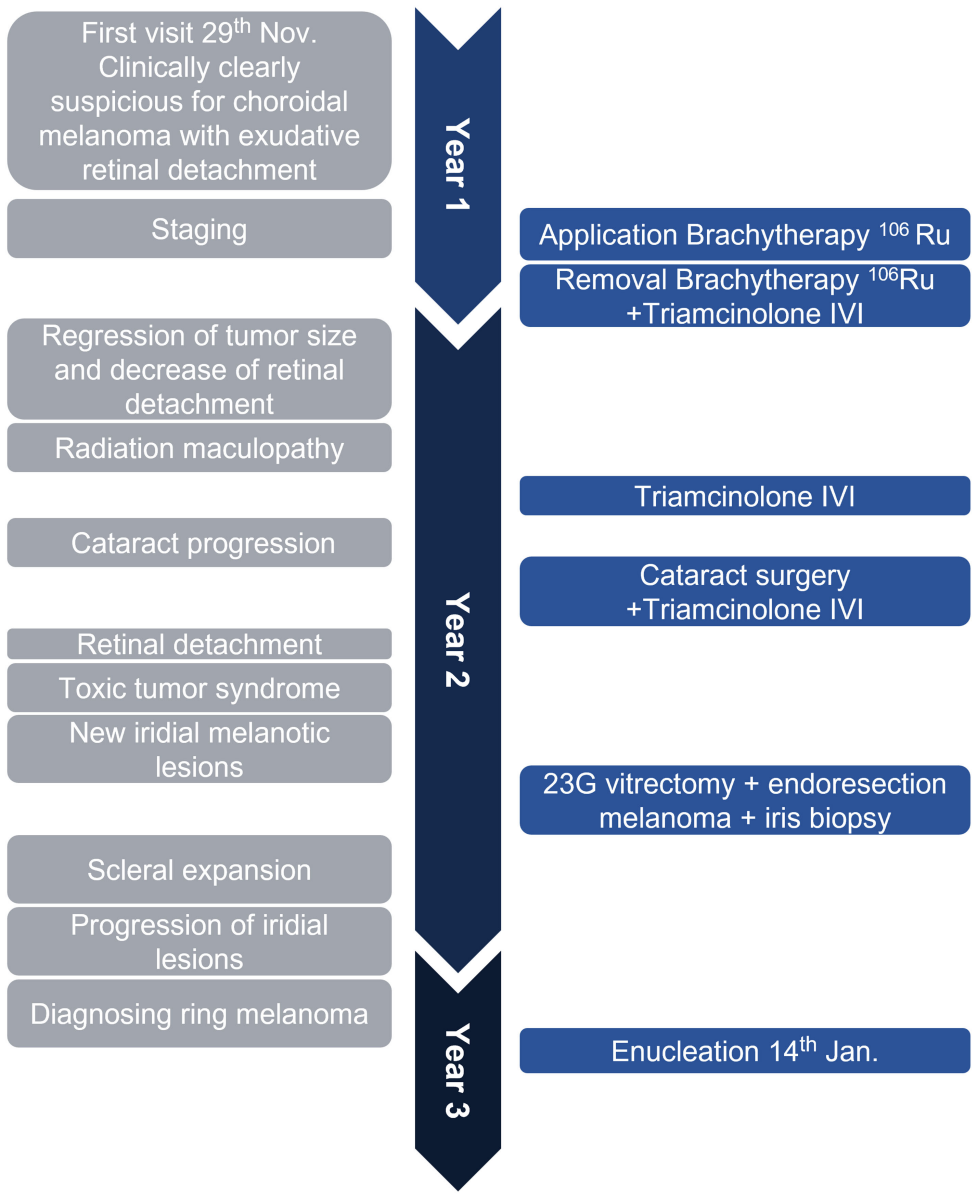
The timeline illustrates the rapid disease course in this case. In about 1 year and 2 months, the patient received the primary diagnosis of uveal melanoma, underwent brachytherapy with good tumor regression, was treated for radiation retinopathy, then diagnosed with a ring-shaped relapse, and underwent enucleation of the eye. The left gray side lists important clinical features, and the right blue side demonstrates surgical interventions. IVI, intravitreal injection; Ru^106^, ruthenium-106; 23G, 23-gauge.

**Figure 4** illustrates and summarizes the chronology of the case.

The patient returns to our clinic for regular follow-up visits, during which we did not detect any signs of a local recurrence until today at 1 year after enucleation.

Over the disease course, the patient had received regular aftercare examinations to exclude metastases, including ultrasound imaging of the abdomen, serum liver enzyme levels, and chest X-rays. There were no suspicious lesions at any time.

## Discussion

Ring melanomas represent a very rare variant of uveal melanomas that are associated with a poor prognosis and often result in the enucleation of the affected eye (2). Detailed epidemiological studies are hampered by the rarity of this entity. Demirci *et al.* studied 8,800 patients with uveal melanoma and detected 23 cases with ciliary body ring melanoma (0.3%). In this study, all of the cases were managed with enucleation (2). It is often impossible to identify the site of primary growth, which can be the ciliary body, the base of the iris, or the iridociliary junction.

To our knowledge, so far, there are only two case descriptions of patients in which a relapse of a treated localized uveal melanoma presented as a ring melanoma. Meyer *et al.* described a patient diagnosed with ring melanoma 3 years after a proton beam treatment for a ciliary body melanoma (5). Tay *et al.* reported a ring-shaped recurrence with extrascleral extension of a ciliary body melanoma after plaque brachytherapy (6). In their case, the tumor relapsed 14 years after brachytherapy, shortly after performing trabeculectomy. In all published cases, it remains unclear whether these findings are real tumor recurrences or subclinical remaining tumor cells in the iridociliary junction.

Our case represents a highly aggressive disease course of uveal melanoma which recurred after brachytherapy. The preoperatively present exudative retinal detachment is a known predictive factor for recurrence after radiation therapy (7, 8). In our patient, after plaque brachytherapy, we were able to observe a very good response with tumor remission until there was no more tumor prominence noticeable on ultrasound. The first signs of a recurrence presented only 1 year after brachytherapy with new melanotic iridial lesions.

Clinically, the differential diagnoses of a multilocular melanoma and a *de novo* melanoma after brachytherapy must be taken into consideration. Since we were able to illustrate a continuous circumferential tumor growth infiltrating the iris and ciliary body in UBM and later in the histopathological assessment, the diagnosis of a ring melanoma can be made. It can be speculated that the partial thickening of the ciliary body that we noticed before brachytherapy was already the beginning of a ring melanoma, which would represent a typical course in ring melanomas (2).

The histopathological and genetic analyses revealed the same characteristics in the treated uveal melanoma, and the latter detected iridial lesions, suggesting the ring melanoma to be most likely a relapse of the primary uveal melanoma. However, it cannot be completely excluded that the ring melanoma formed *de novo*.

Our histopathological analyses revealed an epithelioid cell type, which is a rather rare form of uveal melanoma and is associated with a poorer prognosis (9, 10).

Ring melanomas have not been systematically reviewed concerning their genetic features. However, molecular genetic analyses gain more and more importance as they provide us with information that are of increasing clinical relevance, especially concerning prognosis and personalized therapy options. This case reports a unique genetic pattern that has not been shown in ring melanoma yet.

Utilizing FISH, we detected 23% chromosomal aberrations concerning monosomy 3 (complete loss of one chromosome 3). Monosomy 3 is a significant predictor for a higher relapse rate and lower overall survival rates (11, 12). All analyzed nuclei showed changes of p1, which is a common feature of uveal melanoma (13). The amplification of *MYC*, as in this case, occurs in about 40% of all uveal melanoma (14).

Using next-generation sequencing, we investigated *GNA11*, *GNAQ*, and *SF3B1*. The activating hotspot mutation c.626A>T in *GNAQ*, which leads to an amino acid change from glutamine to leucine, has already been described and occurs in approximately 45% of primary uveal melanomas and 22% of uveal metastases (15). The mutations lead to the constitutive activation of the Gαq and Gα11 subunits by decreasing their GTPase activity, which is required to return them to an inactive state. This results in the constitutive activation of G-protein-coupled signaling pathways, such as MAPK, PI3K, PKC, Akt/mTOR, Rac/Rho, Wnt/β-catenin, and Hippo (13, 16). *GNAQ* mutations occur early in uveal melanoma as tumor-initiating mutations and do not seem to have a prognostic value (17).

Moreover, we were able to find a mutation in *SF3B1* that leads to an amino acid exchange of arginine to histidine (c.1874G>A; p.Arg625His). The mutations of codon 625 in *SF3B1* can be identified in 20% of uveal melanomas (18). They result in an aberrant splicing pattern of important apoptotic genes through the usage of an alternative 3′ splice site upstream of the canonical 3′ splice site (16). The mutation c.1874G>A has so far only been described by Hou *et al.* in 19 of 85 examined uveal melanomas (19). The clinical significance of this variant concerning disease course and prognosis is still unclear. However, information on the mutation status in uveal melanoma are crucial as more and more individual therapeutic concepts are emerging. Since *SF3B1* is involved in splicing, there has been growing interest in splicing modulators. Additionally, *SF3B1* mutations may also be sensitive to nonsense-mediated mRNA decay inhibitors or protein arginine methyltransferase 5 inhibitors (13, 20, 21).

To date, the management options for ring melanomas are still limited. Based on the large tumor dimension, enucleation is usually the preferred treatment option. The tumor in our case has certain characteristics that limit our patient’s prognosis. First, ring melanomas have a poorer prognosis than localized uveal melanomas, with a metastatic rate of 52% after 5 years of follow-up (2). Additionally, ciliary body infiltration, monosomy 3, epithelioid cell type, and extrascleral extension are limiting factors. The patient regularly returns to our clinic and attends aftercare examinations. So far, no signs of a local recurrence or metastatic disease could be detected.

With this case report, we want to emphasize the importance of regular follow-up visits of patients with uveal melanoma, without and with pupil dilation. The interindividual disease course is highly variable, and in cases like this, disease progression and the possibility to intervene could be missed if follow-up intervals are extended. Additionally, we would like to encourage the genetic testing of biopsies of uveal melanoma and especially ring melanoma. Even though this entity is very rare, genetic testing is crucial to gain further insights into the pathomechanisms and could have therapeutic relevance as individualized treatment strategies emerge.

## Concluding Remarks

This case illustrates a particularly aggressive local disease course in a uveal melanoma that relapsed as a ring melanoma. We detected hotspot mutations in *GNAQ* and *SF3B1* that have been described in uveal melanoma, but not ring melanoma. Molecular genetic analyses like in this case provide valuable insights into the disease entity that form the base for future therapeutic concepts as personalized medicine gains more and more importance.

## Data Availability Statement

The original contributions presented in the study are included in the article/supplementary material. Further inquiries can be directed to the corresponding author.

## Ethics Statement

Written informed consent was obtained from the patients/participants for the publication of this case report.

## Author Contributions

JCF, MP, VK, MR, SL, and FR were responsible for the patient during clinical visits. VK and FR performed surgeries. Genetic analyses were performed and evaluated by MS and AC. Histopathological assessment was done by HM. JCF and MP took the microscopic and clinical images and created the figures. MP, VK, MR, and FR wrote the sections of the manuscript. All authors contributed to the article and approved the submitted version.

## Conflict of Interest

The authors declare that the research was conducted in the absence of any commercial or financial relationships that could be construed as a potential conflict of interest.

## Publisher’s Note

All claims expressed in this article are solely those of the authors and do not necessarily represent those of their affiliated organizations, or those of the publisher, the editors and the reviewers. Any product that may be evaluated in this article, or claim that may be made by its manufacturer, is not guaranteed or endorsed by the publisher.
